# Early ART-initiation and longer ART duration reduces HIV-1 proviral DNA levels in children from the CHER trial

**DOI:** 10.1186/s12981-021-00389-1

**Published:** 2021-09-29

**Authors:** Helen Payne, Man K. Chan, Sarah A. Watters, Kennedy Otwombe, Nei-Yuan Hsiao, Abdel Babiker, Avy Violari, Mark F. Cotton, Diana M. Gibb, Nigel J. Klein

**Affiliations:** 1 Institute of Child Health, University College London, London, United Kingdom; 2 Department of Infection and Immunity, University College London, London, United Kingdom; 3 Department of Clinical Microbiology and Virology, University College London Hospitals NHS Trust, London, United Kingdom; 4 Perinatal HIV Research Unit, Faculty of Health Sciences, University of the Witwatersrand, Johannesburg, South Africa; 5 Department of Paediatrics and Child Health, Family Center for Research With Ubuntu, Stellenbosch University, Cape Town, South Africa; 6 Division of Medical Virology, Department of Pathology, University of Cape Town and National Health Laboratory Service, Cape Town, South Africa; 7 MRC Clinical Trials Unit at University College London, London, United Kingdom; 8 Department of Paediatric Infectious Diseases, Imperial College London, Room 235, Medical School Building, Norfolk Place, London, W21PG United Kingdom

**Keywords:** HIV-1 proviral DNA, Children, Reservoir, ART, CHER

## Abstract

**Background:**

Reduction of the reservoir of latent HIV-infected cells might increase the possibility of long-term remission in individuals living with HIV. We investigated factors associated with HIV-1 proviral DNA levels in children receiving different antiretroviral therapy (ART) strategies in the children with HIV early antiretroviral therapy (CHER) trial.

**Methods:**

Infants with HIV  <  12 weeks old with CD4%  ≥  25% were randomized in the CHER trial to early limited ART for 40 or 96 weeks (ART-40 W, ART-96 W), or deferred ART (ART-Def). For ART-Def infants or following ART interruption in ART-40 W/ART-96 W, ART was started/re-started for clinical progression or CD4%  <  25%. In 229 participants, HIV-1 proviral DNA was quantified by PCR from stored peripheral blood mononuclear cells from children who had received  ≥  24 weeks ART and two consecutive undetectable HIV-1 RNA 12–24 weeks apart. HIV-1 proviral DNA was compared between ART-Def and ART-96 W at week 96, and in all arms at week 248. Factors associated with HIV-1 proviral DNA levels were evaluated using linear regression.

**Findings:**

Longer duration of ART was significantly associated with lower HIV-1 proviral DNA at both 96 (p  =  0.0003) and 248 weeks (p  =  0.0011). Higher total CD8 count at ART initiation was associated with lower HIV-1 proviral DNA at both 96 (p  =  0.0225) and 248 weeks (p  =  0.0398). Week 248 HIV-1 proviral DNA was significantly higher in those with positive HIV-1 serology at week 84 than those with negative serology (p  =  0.0042).

**Intepretation:**

Longer ART duration is key to HIV-1 proviral DNA reduction. Further understanding is needed of the effects of “immune-attenuation” through early HIV-1 exposure.

**Funding:**

Wellcome Trust, National Institutes of Health, Medical Research Council.

## Introduction

An estimated 3.3 million children under 15 years of age live with human immunodeficiency virus (HIV) worldwide, over 90% in sub-Saharan Africa [1, 2]. The introduction of antiretroviral therapy (ART) early in life has substantially reduced morbidity and mortality [3, 4] and optimised CD4 cell reconstitution [5]. Although HIV-1 virological suppression is achievable in most children, as in adults, HIV remains latent and integrated within the host genome in subpopulations of infected cells [6]. This reservoir, commonly estimated by quantitative measures of HIV-1 proviral DNA [7, 8], occurs in many cell types including CD34 stem cells, CNS macrophages, astrocytes and dendritic cells [9–11]: however resting CD4 memory cells are considered a critical reservoir due to their longevity, homeostatic cell division and potential for reactivation on antigen encounter [12].

There is increasing interest in viral reservoirs following reports of HIV remission (also known as functional cure) in adults and children treated soon after infection [13]. Remission is defined as lack of detectable virus in blood and a functional immune system without the need for ART, despite detectable HIV using sensitive assays. The case report of the “Mississippi Baby” who received ART from 31 hours of age focused attention on very early ART in children. The mother discontinued ART around 15–18 months of age and when retested at 23 months old, the infant had undetectable plasma HIV-RNA and only traces of proviral DNA just above detectable limits at 24 and 26 months of age. The infant maintained virological suppression for 27.6 months before viral resurgence [14, 15]. One of 227 early-treated children from the children with HIV early antiretroviral therapy (CHER) trial stopped ART after 40 weeks, as per trial randomization, and remained negative for HIV diagnostic tests at the age of 9.5 years [16]. Virus persists at very low levels in plasma and is detectable as low levels of cell-associated DNA, but immunologically he is not unlike healthy children of similar age. There is also a French teenager who received ART from 3 months of age, after a 6 week course of zidovudine was started at birth. After ART was discontinued at approximately 6 years of age, she has remained virologically suppressed for 11 years [17].

Novel approaches to attain HIV remission, including depletion of T-cell subsets known to have integrated HIV-1 DNA, elimination of latent reservoir through activation and clearance mechanisms, and interference with memory CD4 T-cell homeostasis are being pursued [12, 18, 19]. However, as these are not presently considered viable options, early ART initiation remains the therapeutic focus to reduce reservoir size.

Formation and stability of the HIV-1 reservoir in the presence of ART is not well understood [20–22], particularly whether its persistence is primarily due to longevity of latently infected cells or low-level replication [23–25]. Although earliest possible ART initiation is considered optimal in vertically infected children [26–28], it is unclear how timing of initiation, ART duration or ART-interruption within early childhood years, impacts on viral reservoirs and immune responses [29]. Further knowledge in these areas could inform practical approaches towards functional cure; yet even in the absence of a functional cure, the long-term impact of different ART-strategies on reservoir size is of considerable interest with potential to inform future ART management strategies in childhood.

In this sub-study of the CHER trial [3, 4], we compare peripheral HIV-1 proviral DNA in children who received early ART but interrupted ART temporarily versus deferred but continuous ART. We examine the effect of ART-interruption and factors associated with low HIV-1 proviral DNA up until 5 years of age.

## Materials and methods

### Participants

The CHER trial compared early limited ART (zidovudine, lamivudine and lopinavir-ritonavir) for 40 or 96 weeks (ART-40W or ART-96W) with deferred ART (ART-Def) in HIV-infected infants < 12weeks old with baseline CD4 ≥ 25% [3, 4] enrolled between 2005 and 2008. HIV was diagnosed by HIV DNA PCR and confirmed with RNA viral load (VL) > 1000 copies/ml. For infants on deferred ART or following ART interruption after 40 or 96 weeks, ART was started/re-started for clinical progression (protocol-defined CDC severe stage B/C disease) or CD4% < 25% in infants and < 20% in older children.

HIV-1 proviral DNA was measured by quantitative PCR using DNA extracted from 322 samples of cryopreserved peripheral blood mononuclear cells (PBMCs) collected at 12-weekly time-points from 40 to 248 weeks of the trial in 229 participants (Table 1). The use of stored samples was approved by the Human Research Ethics Committees of Stellenbosch University and the University of the Witwatersrand (M12/01/005 and 040703) for the two trial sites: Children’s Infectious Disease Clinical Research Unit (KIDCRU, now the Family Center for Research with Ubuntu) and The Perinatal HIV Research Unit (PHRU). To minimise inclusion of episomal DNA, samples were restricted to those available from all children on ART who were virally suppressed for at least 24 weeks with two consecutive viral loads below 400 copies/ml 12–24 weeks apart [30]. In addition, children must have adhered to the CHER ART-strategies. Using these criteria, all available specimens were used from trial weeks 40 (ART-96W only), weeks 96 (ART-Def and ART-96W) and week 248 (all arms). Children from ART-Def who fulfilled these criteria at 3 or 4 time-points on continuous ART (96, 156, 204, 248 and 252 weeks) were also analysed to illustrate the trajectory of HIV-1 proviral DNA decline on continuous ART. Together these samples were used to answer the following questions: (1) What factors are associated with low levels of HIV-1 proviral DNA at 96 and 248 weeks of ART? (2) What is the effect of early ART followed by interruption on HIV-1 proviral DNA levels at 248 weeks compared to deferred ART without interruption i.e., comparing all CHER ART-strategies: ART-Def, ART-40W and ART-96W?

**Table 1:** PBMC sample availability

	ART-Def, N or N (%)	ART-40 W, N or N (%)	ART-96 W, N or N (%)	Total, N or N (%)
Number of children				
Randomised into the main CHER trial	125	143	143	411
Who died^a^	21	14	12	47
Had samples available^a^	79	56	94	229
Number of samples available				
At week 40			8 (6.5%)	8 (2.4%)
At week 96	44 (29.3%)	–	73 (58.9%)	117 (35.5%)
At week 156	13 (8.7%)	–	–	13 (3.9%)
At week 204	13 (8.7%)	–	–	13 (3.94%)
At week 248	70 (46.7%)	56 (100%)	43 (34.7%)	169 (51.2%)
At week 252	10 (6.7%)	–	–	10 (3.0%)
Total	150	56	124	330

^a^Reasons for samples not included: to compare HIV-1 proviral DNA levels between early and deferred ART at 96 weeks: 81 ART-Def and 70 ART-96 W samples were not tested because the child had died (21 vs 12), samples were not stored (10 vs 6), was not on ART (5 vs 0), or was on ART but not virally suppressed or insufficient evidence of being virally suppressed i.e.,  <  2 plasma samples of  <  400 copies/ml of HIV-1 RNA 12–24 weeks apart (45 vs 54). To compare HIV-1 proviral DNA levels across all three arms at 248 weeks: 55 ART-Def, 87 ART-40 W and 100 ART-96 W samples were not analysed because the child had died (21 vs 14 vs 12), was not on ART (0 vs 29 vs 45), was on ART but not virally suppressed (4 vs 12 vs 4), samples were not stored (30 vs 23 vs 14), or the child did not adhere to CHER ART-strategy i.e. did not interrupt ART when scheduled (0 vs 9 vs 25)

We explored the relationship between HIV-1 proviral DNA and clinical and immunological characteristics available from the CHER trial, including baseline (randomisation) viral loads, CMV serostatus and quantification at randomisation, and HIV-1 serostatus and quantitative HIV-specific antibodies (anti-gp120 IgG at week 84 in ART-Def and ART-96W [31]) at trial week 84. Week 84 was the closest available timepoint to week 96 where serum samples were available for analysis from children on ART [31].

### DNA extraction of PBMCs

PBMCs were isolated from whole blood using standard Ficoll separation and cryopreserved in liquid nitrogen in 10% dimethyl sulfoxide and 90% fetal calf serum. Cryopreserved PBMCs were thawed to room temperature and DNA extracted using the QIAGEN^®^ QIAmp DNA extraction kit (Hilden, Germany). Extracted DNA was eluted from the mini spin column, quantified on the nanodrop (Thermo Scientific, Massachusetts, USA), and stored at – 20 °C until PCR.

### Quantification of total HIV-1 DNA

As described by Smith et al. [32], primers and probe were used to detect total HIV-1 DNA by amplifying the region between LTR and gag. Additionally, primers and probe for human pyruvate dehydrogenase (PDH) were duplexed in the reaction as an internal control. A standard curve was generated using a 6-point logarithmic scale of DNA extracted from 8E5 cells [33] (ATCC), which contain one copy of HIV provirus per cell.

HIV-1 proviral DNA was quantified by real-time PCR using Applied Biosystems 7900HT Fast Real-Time PCR System (TaqMan, Life technologies). For each 25 μl PCR reaction the assay included 12.5 μl of QIAgen Multiplex PCR Master Mix, 0.25 μl of each primer PDH or LTR (concentration 10 μM or 20 μM), and 0.25 μl of each PDH and LTR probe at a concentration of 10μM. PCR conditions were 95°C for 15 minutes, then 45 cycles of 94 °C and 60 °C for 1 minute each. To maximize assay sensitivity, 600 ng of extracted DNA from patient samples was added to each reaction well, with both samples and standards run in triplicate. The lowest limit of detection was 10 proviral copies per 10^6^ PBMCs. Undetectable measures of proviral DNA were repeated for verification.

### Statistical analysis

HIV-1 proviral DNA levels were log to base 10 transformed to approximate normality. Factors associated with log_10_ HIV-1 proviral DNA levels at weeks 96 and 248 were investigated using linear regression. Regression diagnostics were examined to ensure that all model assumptions were met.

Factors investigated included age at ART start, birthweight, sex, duration of ART by weeks 96 and 248, CDC stage, initial ART regimen, baseline VL and immunological data (CD4% and count, CD8% and count), time to initial VL suppression, CMV serology, Prevention of mother to child transmission (PMTCT) received, HIV-specific antibody (anti-gp120 IgG) and HIV-1 serology measured at week 84. Variables significantly associated with log_10_ HIV-1 proviral DNA levels at p < 0.10 or plausible clinical factors such as CD8 count at week 248, were included in a multivariable model, using backwards stepwise elimination (exit probability p = 0.05) to reach the final model. Differences between two and three groups were tested using the Wilcoxon rank-sum and Kruskal-Wallis rank tests, respectively. Stata version 15.1 (Stata Corporation, College Station, Texas, USA) was used for all analyses.

## Results

**Fig. 1:**
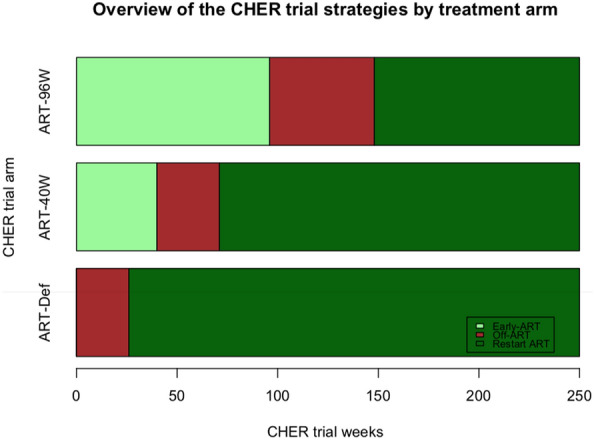
Overview of the CHER trial treatment strategies and median duration on ART within arms from participants analysed for HIV proviral DNA. Median time to ART initiation in the deferred arm, ART-Def, was 26.1 weeks (IQR 20–42.9, n  =  79). Median duration of ART-interruption for ART-40 W was 31.3 (12.5–54.3, n  =  56) and 52.3 (11.4–152, n  =  94) weeks in ART-96 W

To compare HIV-1 proviral DNA levels between early and deferred ART we analysed 44 PBMC samples from 125 children randomised to ART-Def (35.2%), and 73 samples from 143 children in ART-96W (51%) at 96 weeks. To compare HIV-1 proviral DNA levels across all three arms we analysed 70 PBMC samples from 125 children randomised to ART-Def (56%), 56 samples from 143 children from ART-40W (39%), and 43 samples from 143 children from ART-96W (30%) at 248 weeks (Table 1). Figure 1 illustrates an overview of the CHER trial treatment strategies and median duration on ART within arms. Patient characteristics and follow-up immunology, virological suppression and reservoir size at all measured time-points are described in Tables 2, 3 respectively.

**Table 2:** Patient characteristics

	ART-Def, Median (IQR) [N] or N (%)	ART-40 W, Median (IQR) [N] or N (%)	ART-96 W, Median (IQR) [N] or N (%)	Total, Median (IQR) [N] or N (%)
Number of patients	79	56	94	229
Age at randomisation (weeks)	7.3 (6.4, 9.1) [79]	7.9 (7.3, 9.2) [56]	7.6 (6.7, 8.6) [94]	7.6 (6.7, 9.0) [229]
Age at ART start (weeks)	26.1 (20.0, 42.9) [79]	7.9 (7.3, 9.2) [56]	7.6 (6.7, 8.6) [94]	8.7 (7.4, 20.1) [229]
Birthweight (kg)	2.9 (2.6, 3.3) [79]	2.9 (2.6, 3.2) [56]	3.0 (2.7, 3.3) [94]	2.9 (2.6, 3.3) [229]
Gender				
Female	49 (62.0%)	28 (50.0%)	52 (55.3%)	129 (56.3%)
Male	30 (38.0%)	28 (50.0%)	42 (44.7%)	100 (43.7%)
Ethnic origin				
Black	79 (100.0%)	52 (92.9%)	89 (94.7%)	220 (96.1%)
Non-black	0 (0.0%)	4 (7.1%)	5 (5.3%)	9 (3.9%)
Infant PMTCT				
No	9 (11.4%)	5 (8.9%)	14 (14.9%)	28 (12.2%)
Yes	67 (84.8%)	49 (87.5%)	77 (81.9%)	193 (84.3%)
Unknown	3 (3.8%)	2 (3.6%)	3 (3.2%)	8 (3.5%)
Mother PMTCT				
No	10 (12.7%)	6 (10.7%)	10 (10.6%)	26 (11.4%)
Yes	69 (87.3%)	50 (89.3%)	84 (89.4%)	203 (88.6%)
Clinical site				
KIDCRU	24 (30.4%)	26 (46.4%)	29 (30.9%)	79 (34.5%)
PHRU	55 (69.6%)	30 (53.6%)	65 (69.1%)	150 (65.5%)
CDC stage				
A	6 (7.6%)	19 (33.9%)	13 (13.8%)	38 (16.6%)
B	4 (5.1%)	1 (1.8%)	6 (6.4%)	11 (4.8%)
N	69 (87.3%)	36 (64.3%)	75 (79.8%)	180 (78.6%)
Cytomegalovirus serology				
Negative	49 (62.0%)	37 (66.1%)	64 (68.1%)	150 (65.5%)
Positive	10 (12.7%)	11 (19.6%)	18 (19.1%)	39 (17.0%)
No sample/missing	20 (25.3%)	8 (14.3%)	12 (12.8%)	40 (17.5%)
Viral load (copies/ml)				
At randomisation	7,50,001 (5,01,000; 7,50,001) [79]	5,57,500 (1,94,000; 7,50,001) [56]	7,50,001 (6,45,000; 7,50,001) [94]	7,50,001 (4,39,000; 7,50,001) [229]
At ART start	7,50,001 (1,42,400; 7,50,001) [78]	5,57,500 (1,94,000; 7,50,001) [56]	7,50,001 (6,45,000; 7,50,001) [94]	7,50,001 (3,38,000; 7,50,001) [228]
Log_10_ viral load (copies/ml)				
At randomisation	5.9 (5.7, 5.9) [79]	5.7 (5.3, 5.9) [56]	5.9 (5.8, 5.9) [94]	5.9 (5.6, 5.9) [229]
At ART start	5.9 (5.2, 5.9) [78]	5.7 (5.3, 5.9) [56]	5.9 (5.8, 5.9) [94]	5.9 (5.5, 5.9) [228]
Immunology				
CD4%				
At screening	37 (31, 42) [78]	34 (29, 40) [56]	36 (31, 42) [94]	36 (31, 42) [228]
At randomisation	35 (29, 39) [75]	35 (30, 41) [54]	34 (29, 38) [89]	35 (29, 39) [218]
At ART start	22 (19, 30) [78]	35 (30, 41) [54]	34 (29, 38) [89]	31 (25, 37) [221]
CD4 count				
At screening	2543 (1771, 3062) [78]	1955 (1405, 2431) [56]	2242 (1661, 2995) [94]	2225 (1611, 2986) [228]
At randomisation	1968 (1572, 2542) [75]	1918 (1279, 2555) [54]	2002 (1480, 2762) [89]	1956 (1446, 2688) [218]
At ART start	1079 (712, 1495) [78]	1918 (1279, 2555) [54]	2002 (1480, 2762) [89]	1574 (1103, 2425) [221]
CD8%				
At screening	23 (21, 34) [77]	28 (20, 34) [54]	26 (21, 33) [93]	26 (21, 34) [224]
At randomisation	25 (21, 33) [78]	28 (22, 34) [54]	27 (22, 34) [93]	27 (21, 34) [225]
At ART start	34 (29, 43) [79]	28 (22, 34) [54]	27 (22, 34) [93]	30 (23, 37) [226]
CD8 count				
At screening	1589 (1153, 2480) [77]	1494 (1050, 2026) [54]	1648 (1142, 2243) [93]	1597 (1135, 2238) [224]
At randomisation	1416 (1115, 2227) [78]	1331 (998, 2025) [54]	1686 (1139, 2386) [93]	1471 (1094, 2258) [225]
At ART start	1465 (874, 2454) [79]	1331 (998, 2025) [54]	1686 (1139, 2386) [93]	1530 (1036, 2294) [226]

*PMTCT* prevention of mother to child transmission

**Table 3:** Follow-up

	ART-Def, Median (IQR) [N] or N (%)	ART-40 W, Median (IQR) [N] or N (%)	ART-96 W, Median (IQR) [N] or N (%)	Total, Median (IQR) [N] or N (%)
Duration of ART (weeks)^a,b^				
By week 96	84 (72, 84) [44]	–	96 (96, 96) [73]	96 (84, 96) [117]
By week 248	228 (211, 240) [70]	220 (196, 235) [56]	216 (168, 247) [43]	220 (200, 240) [169]
Duration of viral suppression (weeks)^c^				
By week 248	200 (163, 214) [70]	163 (110, 192) [56]	164 (108, 200) [43]	179 (132, 204) [169]
Anti-gp120 IgG at week 84	5208 (812; 24,751) [60]	–	218 (133,662) [87]	420 (169; 13,129) [147]
HIV-1 serology at week 84				
Negative	8 (10.1%)	0 (0%)	41 (43.6%)	49 (21.4%)
Positive	52 (65.8%)	0 (0%)	44 (46.8%)	96 (41.9%)
Not determined	19 (24.1%)	56 (100%)	9 (9.6%)	84 (36.7%)
CD4%				
At week 96	35 (31, 40) [44]	31 (29, 34) [2]	37 (31, 42) [72]	37 (31, 41) [118]
At week 248	36 (33, 40) [70]	34 (29, 39) [54]	32 (27, 36) [43]	35 (30, 39) [167]
CD4 count (cells/μl)				
At week 96	1650 (1244, 2227) [44]	1696 (1595, 1797) [2]	1647 (1131, 2043) [72]	1650 (1190, 2063) [118]
At week 248	1175 (885, 1562) [70]	1110 (866, 1349) [54]	1039 (755, 1374) [43]	1115 (866, 1428) [167]
HIV-1 DNA (copies/10^6^ PBMC)				
At week 40	–	–	317 (44, 884) [8]	317 (44, 884) [8]
At week 96	2415 (499, 7450) [44]	–	325 (53, 3670) [73]	681 (106, 5580) [117]
At week 156	2290 (513; 13,700) [13]	–	–	2290 (513; 13,700) [13]
At week 204	625 (175, 2140) [13]	–	–	625 (175, 2140) [13]
At week 248	1165 (167; 10,900) [70]	4165 (294; 26,150) [56]	915 (172; 15,400) [43]	1220 (184; 17,300) [169]
At week 252	58 (18, 129) [10]	–	–	58 (18, 129) [10]

^a^Duration of ART by w96 was determined by calculating time from ART start to w96 HIV DNA measurement i.e., duration of ART by w96 (Arm 1/3)  =  [w96 HIV DNA date − ART start date] days

^b^Duration of ART by w248 was determined as follows:

•→Arm 1: calculating time from ART start to w248 HIV DNA measurement i.e., duration of ART by w248 (Arm 1)  =  [w248 HIV DNA date − ART start date] days.

•→Arms 2 and 3: summing the time from ART start to ART interruption and time from ART restart to w248 HIV DNA measurement, i.e., duration of ART w248 (Arm 2/3)  =  [ART interruption date − ART start date]  +  [w248 HIV DNA date − ART restart date] days.

^c^Duration of VL suppression by w248 was calculated by summing all the periods of time VL  <  400 copies/ml. Single isolated VL spikes (i.e., VL  ≥  400 copies/ml) preceded and followed by VL  <  400 copies/ml were allowed and counted as VL suppressed

### Factors associated with low levels of HIV-1 proviral DNA

**Fig. 2:**
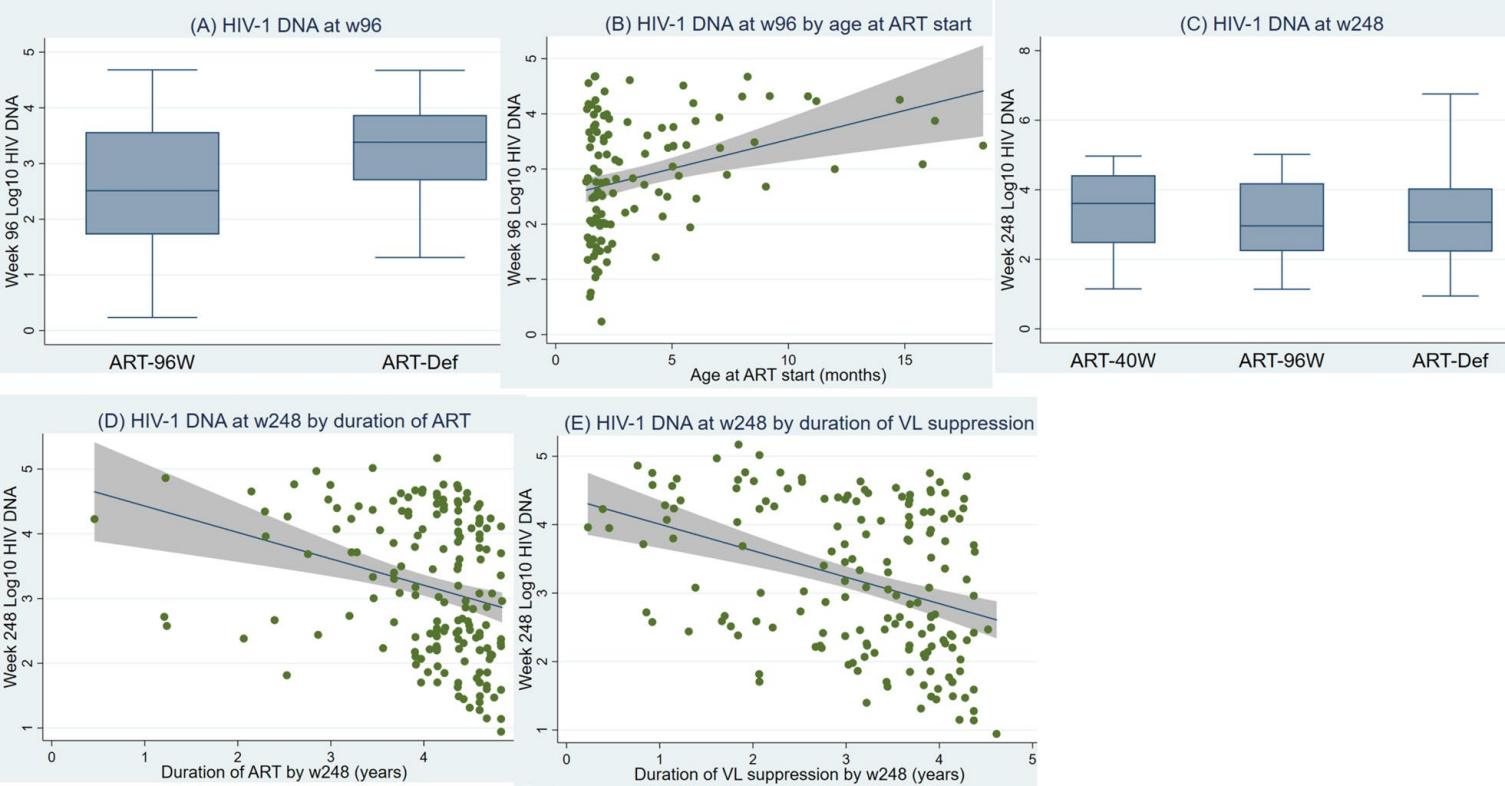
HIV-1 proviral DNA analysis from weeks 96 and 248 of the CHER trial. Y-axes represent HIV-1 proviral DNA (log_10_ copies per 10^6^ PBMCs). Plot A: HIV-1 proviral DNA in ART-Def [n  =  44, median 2415 (IQR 499–7450)] and ART-96 W (n  =  73, median [325 (53–3670) copies of HIV-1 proviral DNA/10^6^ PBMCs] at week 96 (p  =  0.0019). Plot B: HIV-1 proviral DNA at week 96 by age of starting ART (n  =  117). Plot C: HIV-1 proviral DNA in ART-Def (n  =  70, ART-40 W (n  =  56) and ART-96 W (n  =  43) at 248 weeks (Kruskal–Wallis test p  =  0.2553). Plot D: HIV-1 proviral DNA at week 248 by duration of ART received by 248 weeks. Plot E: HIV-1 proviral DNA at trial week 248 by weeks of continuous HIV-1 RNA suppression below 400 copies/ml

Time spent on ART by week 96 was significantly shorter in ART-Def [median 84 (IQR 72–84) weeks] versus ART-96W [96 (IQR 96–96), p < 0.0001]. There was significantly more HIV-1 proviral DNA in ART-Def at 96 weeks [median 2415 (IQR 499–7450)] than ART-96W [325 (53–3670) copies of HIV-1 proviral DNA/10^6^ PBMCs, p = 0.0019, Fig. 2A]. Figure 2B illustrates increasing reservoir size by age of initiating ART, and the range of HIV-1 proviral DNA at ART initiation. In multivariable analysis (Table 4a), at 96 weeks, longer duration of ART was significantly associated with lower levels of HIV-1 proviral DNA [ß = − 1.21 (95% CI − 1.85, − 0.57), p = 0.0003]. This suggests a reduction in HIV-1 proviral DNA percentage by 70% for every further year on ART. The same effect is evident at 248 trial weeks (Table 4b) whereby a reduction of 30% HIV-1 proviral DNA is seen for every further year on ART [ß = − 0.36 (95% CI − 0.15, − 0.15), p = 0.0011].

**Table 4:** Factors associated with HIV-1 DNA levels at weeks 96 and 248

	Univariable model			Multivariable model		
	% change in HIV DNA	β (95% CI)	P-value	% change in HIV DNA	β (95% CI)	P value
**a) Factors associated with HIV-1 DNA at week 96**						
At ART initiation						
Age (per month older)	11.12	0.11 (0.05, 0.16)	0.0002	–	–	–
Log_10_ viral load (copies/ml) (per 10% increase)	− 2.27	− 0.23 (− 0.59, 0.13)	0.2145			
Viral load						
< 7,50,000 copies/ml (ref.: ≥ 7,50,000)	12.57	0.12 (− 0.27, 0.51)	0.5504			
Time to VL suppression (per month longer)	2.30	0.02 (− 0.05, 0.10)	0.5493			
Birthweight (per kg higher)	18.65	0.17 (− 0.29, 0.63)	0.4629			
CD4 count (per 500 cell increase)	− 7.63	− 0.08 (− 0.16, 0.00)	0.0549	–	–	–
CD4% (per 10% increase)	− 10.90	− 0.12 (− 0.33, 0.10)	0.2845			
CD8 count (per 500 cell increase)	− 7.19	− 0.07 (− 0.14, − 0.01)	0.0346	− 7.28	− 0.08 (− 0.14, − 0.01)	**0.0225**
CD8% (per 10% increase)	2.83	0.03 (− 0.14, 0.20)	0.7471			
Gender						
Male (ref.: female)	− 6.62	− 0.07 (− 0.46, 0.32)	0.7282			
CDC stage						
A (ref.: N)	− 13.69	− 0.15 (− 0.77, 0.48)	0.6414	− 2.51	− 0.03 (− 0.61, 0.56)	0.9319
B (ref.: N)	− 60.08	− 0.92 (− 1.72, − 0.12)	0.0247	− 56.64	− 0.84 (− 1.58, − 0.09)	**0.0287**
Site						
KIDCRU (ref.: PHRU)	6.64	0.06 (− 0.36, 0.49)	0.7660			
Cytomegalovirus serology						
Positive (ref.: negative)	− 9.13	− 0.10 (− 0.59, 0.40)	0.7005			
No sample/missing (ref.: negative)	8.38	0.08 (− 0.44, 0.60)	0.7586			
Child PMTCT						
Yes (ref.: no)	19.45	0.18 (− 0.40, 0.76)	0.5438			
Unknown (ref.: no)	8.92	0.09 (− 1.09, 1.26)	0.8856			
Mother PMTCT						
Yes (ref.: no)	− 5.09	− 0.05 (− 0.69, 0.58)	0.8705			
At week 84						
Log_10_ anti-gp120 IgG (per 10% increase)	1.77	0.18 (− 0.01, 0.36)	0.0612	–	–	–
HIV-1 serology						
Positive (ref.: negative)	37.79	0.32 (− 0.10, 0.74)	0.1298			
Not determined (ref.: negative)	67.56	0.52 (− 0.22, 1.25)	0.1654			
Duration of ART by week 96 (per year longer)	− 70.15	− 1.21 (− 1.87, − 0.55)	0.0004	− 70.14	− 1.21 (− 1.85, − 0.57)	**0.0003**
**b) Factors associated with HIV-1 DNA at week 248**						
At ART initiation						
Age (per month older)	1.16	0.01 (− 0.02, 0.04)	0.4675			
Log_10_ viral load (copies/ml) (per 10% increase)	1.42	0.14 (− 0.14, 0.43)	0.3273			
Viral load						
< 7,50,000 copies/ml (ref.: ≥ 7,50,000)	− 15.38	− 0.17 (− 0.54, 0.21)	0.3803			
Time to VL suppression (per month longer)	0.34	0.00 (− 0.07, 0.07)	0.9232			
Birthweight (per kg higher)	− 21.08	− 0.24 (− 0.62, 0.15)	0.2283			
CD4 count (per 500 cell increase)	4.93	0.05 (− 0.05, 0.14)	0.3223			
CD4% (per 10% increase)	14.99	0.14 (− 0.05, 0.33)	0.1505			
CD8 count (per 500 cell increase)	− 5.20	− 0.05 (− 0.13, 0.02)	0.1556	− 6.97	− 0.07 (− 0.14, − 0.00)	**0.0398**
CD8% (per 10% increase)	− 15.01	− 0.16 (− 0.32, − 0.00)	0.0489	–	–	–
Gender						
Male (ref.: female)	− 21.86	− 0.25 (− 0.62, 0.13)	0.1940			
CDC stage						
A (ref.: N)	− 30.85	− 0.37 (− 0.85, 0.11)	0.1286			
B (ref.: N)	− 30.97	− 0.37 (− 1.16, 0.42)	0.3567			
Site						
KIDCRU (ref.: PHRU)	− 35.52	− 0.44 (− 0.81, − 0.06)	0.0219	− 40.25	− 0.52 (− 0.85, − 0.18)	**0.0031**
Cytomegalovirus serology						
Positive (ref.: negative)	− 19.03	− 0.21 (− 0.74, 0.31)	0.4291			
No sample/missing (ref.: negative)	31.86	0.28 (− 0.21, 0.77)	0.2667			
Child PMTCT						
Yes (ref.: no)	− 39.28	− 0.50 (− 1.03, 0.03)	0.0658	–	–	–
Unknown (ref.: no)	− 25.04	− 0.29 (− 1.39, 0.81)	0.6050	–	–	–
Mother PMTCT						
Yes (ref.: no)	− 13.90	− 0.15 (− 0.70, 0.40)	0.5944			
At week 84						
Log_10_ anti-gp120 IgG (per 10% increase)	3.08	0.31 (0.07, 0.55)	0.0131	–	–	–
HIV-1 serology						
Positive (ref.: negative)	64.24	0.50 (− 0.08, 1.07)	0.0911	106.59	0.73 (0.23, 1.22)	**0.0042**
Not determined (ref.: negative)	60.24	0.47 (− 0.09, 1.03)	0.0981	90.99	0.65 (0.16, 1.13)	**0.0093**
Duration of ART by week 248 (per year longer)	− 36.27	− 0.45 (− 0.68, − 0.22)	0.0002	− 30.01	− 0.36 (− 0.57, − 0.15)	**0.0011**
Duration of VL suppression by week 248 (per year longer)	− 30.16	− 0.36 (− 0.53, − 0.19)	4.4E-05	–	–	–

Factors that remained significant *p* < 0.05 are in bold

At w96, HIV-1 DNA was measured in 44 ART-Def and 73 ART-96 W children. At w248, HIV-1 DNA was measured in 70 ART-Def, 56 ART-40 W and 43 ART-96 W arms. Factors significantly associated with HIV-1 DNA in univariable analyses were included in the multivariable (adjusted) analysis

Age at ART start was not fitted in the multivariable model due to multicollinearity with duration of ART by w96

Duration of VL suppression by w248 was not fitted in the multivariable model due to multicollinearity with duration of ART by w248

Not determined: these are samples not tested including ART-40 W and some not tested in ART-Def and ART-90 W (see Table 3 for numbers)

*VL* viral load; *CDC* Centre for Disease Control; *KIDCRU* Children’s Infectious Diseases Clinical Research Unit, Cape Town; *PHRU* Perinatal HIV Research Unit, Johannesburg; *PMTCT* Prevention of mother to child transmission; *β* regression coefficient estimates; *95% CI* 95% confidence interval of β; *% change in HIV-1 DNA* a unit change in factors investigated is associated with a Y% change in HIV-1 DNA, e.g., a month increase in age at ART start is associated with a 11.12% increase in HIV-1 DNA (univariable model)

Higher total CD8 count at ART initiation was associated with lower HIV-1 proviral DNA at both 96 and 248 weeks [ß = − 0.08 (95% CI − 0.14, − 0.01), p = 0.0225 and ß = − 0.07 (95% CI − 0.14, − 0.00), p = 0.0398, respectively]. Therefore, for every 500 cell increase in total CD8 count, a reduction of approximately 7% in HIV-1 proviral DNA was demonstrated at weeks 96 and 248.

Compared to CDC stage N at enrolment, CDC stage B was associated with a 57% reduction in HIV-1 proviral DNA levels at 96 weeks [ß=− 0.84 (95% CI − 1.58, − 0.09), p = 0.0287]. This relationship was not seen at 248 weeks. There was 40% more reduction of HIV-1 proviral DNA levels at KIDCRU, the Cape Town trial study site compared to PHRU, the Johannesburg study site [ß = − 0.52 (95% CI − 0.85, − 0.18), p = 0.0031] at 248 weeks (but not 96 weeks). Multivariable analysis did not suggest that the different maternal or child PMTCT used at the two trial sites significantly affected total HIV-1 proviral DNA at either week 96 or 248, however cumulative time on ART at 248 weeks was significantly shorter in participants from PHRU [mean 204, median 220 (IQR 184–233)] versus KIDCRU [mean 220, median 220 (IQR 208–240) weeks, Wilcoxon rank-sum test p = 0.042].

At 248 weeks, HIV-1 proviral DNA levels were significantly higher in those with positive HIV-1 serology determined at 84 weeks compared to children with negative HIV-1 serology [ß = 0.73 (95% CI 0.23, 1.22), p = 0.0042]. The same relationship was evident between undetermined serology (not determined in 56 ART-40W, 9 ART-96W and 19 ART-Def children) and increased HIV-1 proviral DNA levels compared with negative HIV-1 serology. Age at ART start was not fitted in the multivariable model due to multicollinearity with duration of ART by week 96; and duration of viral load suppression by week 248 was not fitted in the multivariable model due to multicollinearity with duration of ART by week 248.

### Effect of time-off ART on HIV-1 proviral DNA

The effect of time off ART in the first (ART-Def, n = 70), second (ART-40W, n = 56) or third (ART-96W, n = 43) year of life was assessed by comparing HIV-1 proviral DNA between the treatment strategies at 248 weeks. In the children examined, the total time spent on ART from enrolment until 248 weeks was higher in ART-Def [median 228 (IQR 211, 240) weeks, 220 (196, 235) for ART-40W, 216 (168, 247) for ART-96W; p = 0.0001], as was the duration of viral load suppression [median 200 (IQR 163, 214) weeks for ART-Def, 163 (110, 192) for ART-40W, 164 (108, 200) for ART-96W; p = 0.0001]. There was no significant difference at 248 weeks between the 3 arms in HIV-1 proviral DNA [median 1165 (IQR 167; 10,900) HIV-1 proviral DNA/10^6^ PBMCs for ART-Def, 4165 (294; 26,150) for ART-40W, 915 (172; 15,400) for ART-96W; p = 0.2553, Fig. 2C].

Figure 2D, E illustrate the spread of the data and how some individuals have high HIV-1 proviral DNA despite long duration of ART or viral load suppression. Of the 229 children studied, only 2 children had HIV-1 proviral DNA measurements ≤ 50 copies/10^6^ PBMCs: 1 child from ART-Def with undetectable proviral DNA at 252 weeks and 1 child from ART-96W with HIV-1 proviral DNA 6 copies/10^6^ PBMCs at 96 weeks.

## Discussion

Our study is currently the largest analysis of HIV-1 proviral DNA data from a randomized controlled trial in children and demonstrated that HIV-1 proviral DNA was significantly lower after 96 weeks of ART in children who started early ART than those with ART deferred until clinically or immunologically indicated. In multivariable analyses, longer duration of ART was significantly associated with lower levels of HIV-1 proviral DNA at both weeks 96 and 248, thus supporting the results from similar but smaller studies [34–41].

The effect of duration of HIV infection and age of ART initiation on HIV-1 proviral DNA levels cannot be distinguished, since these two variables are intrinsically related. Also, it is not possible to determine the exact timing of infection, which can be either in utero, intrapartum or after birth. There is an inherent selection bias as more children died in ART-Def than early treated arms, none of whom had samples available for evaluation. Clinically unwell children may also have been less likely to have an aliquot of PBMCs or plasma stored. However, these biases most likely underestimate the associations seen as unwell children are more likely to have uncontrolled HIV-1 with potentially higher proviral DNA levels. This is reflected by the fact that the greater reduction in HIV-1 proviral DNA in children with CDC Stage B at ART initiation may be due to having higher levels of integrated HIV-1 DNA before starting ART due to more advanced disease. The assay used measured total HIV-1 proviral DNA, i.e. both integrated and episomal LTR, therefore it was essential that children were virally suppressed for at least 12 weeks and it was not possible to measure baseline proviral DNA in these children.

There was no significant difference in HIV-1 proviral DNA at 248  between the 3 trial arms, suggesting the beneficial effect on reservoir reduction through early ART may be lost with treatment interruption, as reflected by other smaller studies [42, 43]; yet equally how continuous ART may counteract the detrimental effect of delayed ART initiation compared to early-ART followed by interruption. Subsequent analysis from a small group of children who did not interrupt early ART in the CHER trial demonstrated lower cell-associated HIV-1 DNA and RNA at 7–8 years of age in those that started ART before 2 months of age compared to after 2 months of age [41]. Furthermore, initiation of continuous ART within the first week of life in another group of HIV-infected infants [43, 44] has been associated with more rapid HIV-1 DNA decay compared to ART-initiation at median of 7 weeks of age followed by ART-interruption (ART-96W) and ART-initiation at median 22 weeks followed by continuous ART (ART-Def). These data, and other recent studies support earlier initiation and sustained virological suppression within the first 2 years of life to most effectively reduce reservoir size [27, 28, 45, 46].

HIV-1 seropositivity at 84 weeks was associated with higher proviral DNA at 248 weeks, however quantitative HIV-1 anti-gp120 antibody levels did not show this relationship [31], although when applying a wider range of HIV-specific antibodies has since demonstrated to estimate reservoir size [47]. While duration of ART is clearly a key determinant of the viral reservoir, we observed individual children with high HIV-1 proviral DNA levels despite long-term ART and RNA suppression. This has been described previously [48, 49] and might be explained by longer periods of intra-uterine infection, and possibly interval viral load testing not capturing “viral blips”. It may also be due to homeostatic proliferation of HIV-infected cells in the absence of viral reactivation [50, 51], although this has not been demonstrated in peripheral blood mononuclear cells [52]. Despite virological control with ART, persistent inflammation and immune activation is recognized in perinatally-acquired HIV [53, 54], potentially driving proliferation of cells that harbor latent integrated HIV [12] such as within follicular B-cells [55, 56].

Higher CD8 count at ART initiation was associated with a greater reduction in HIV-1 proviral DNA. This may be due to the proportion of CD8 T-cells in the PBMCs from which DNA was extracted; or reflect a functional immune response to HIV-infection, implying that the dynamics of reservoir decline may not be solely reliant on adequate viral suppression [57]. In response to HIV, total levels of CD8 increase from a combination of thymic output of naïve CD8 T-cells and proliferation via clonal expansion. This may be regarded as “immune-attenuation” and suggests that alongside ART, CD8 T-cells may play an important role in controlling HIV infection and potentially mediate eradication of viral reservoirs of infection through interaction with various HLA types such as HLA-B*27:02 [58]. The vast majority of children had viral loads from enrolment reported as > 7,50,000 copies/ml therefore we are unable to determine whether a relationship exists between higher viral loads and higher CD8 counts.

A site effect was observed as children at KIDCRU had significantly lower levels of HIV-1 proviral DNA at week 248 than at PHRU. This may reflect variation in clinician approach to starting or re-starting ART, as reflected by increased time on ART in KIDCRU participants compared to PHRU.

## Conclusions

Our study affirms the association between earlier initiation and longer duration of ART and reduced levels of HIV-1 proviral DNA in children. We also demonstrated associations between higher CD8 count at ART initiation with greater reduction of HIV-1 proviral DNA, inferring a possible beneficial effect of early HIV-1 viral exposure and “immune-attenuation” alongside ART. While we have analysed multiple factors, there are other factors that have not been examined that may plausibly influence levels of HIV-1 proviral DNA: timing of HIV-transmission and initial maternal viral burden, ART adherence, co-infections, thymic output and immune activation. Further work is required to better understand these dynamics and identify potential targets for adjunctive HIV-1 reduction strategies, now a major approach for reducing HIV reservoirs as children become adults.

## Data Availability

The datasets used and/or analysed during the current study are available from the corresponding author on reasonable request.
